# Do gliosarcomas have distinct imaging features on routine MRI?

**DOI:** 10.1177/19714009211012345

**Published:** 2021-04-30

**Authors:** Christoph J Maurer, Irina Mader, Felix Joachimski, Ori Staszewski, Bruno Märkl, Horst Urbach, Roland Roelz

**Affiliations:** 1 Department of Diagnostic and Interventional Radiology and Neuroradiology, University Hospital Augsburg, Germany; 2 Department of Neuroradiology, Medical Center, University of Freiburg, Germany; 3 Department of Radiology, Schön-Klinik, Germany; 4 Institute of Neuropathology, Faculty of Medicine, University of Freiburg, Germany; 5 Institute of Pathology, University Hospital Augsburg, Germany; 6 Department of Neurosurgery, Faculty of Medicine, University of Freiburg, Germany

**Keywords:** Gliosarcoma, MRI, logistic regression model, multivariate analysis, glioblastoma

## Abstract

**Purpose:**

The aim of this study was the development and external validation of a logistic regression model to differentiate gliosarcoma (GSC) and glioblastoma multiforme (GBM) on standard MR imaging.

**Methods:**

A univariate and multivariate analysis was carried out of a logistic regression model to discriminate patients histologically diagnosed with primary GSC and an age and sex-matched group of patients with primary GBM on presurgical MRI with external validation.

**Results:**

In total, 56 patients with GSC and 56 patients with GBM were included. Evidence of haemorrhage suggested the diagnosis of GSC, whereas cystic components and pial as well as ependymal invasion were more commonly observed in GBM patients. The logistic regression model yielded a mean area under the curve (AUC) of 0.919 on the training dataset and of 0.746 on the validation dataset. The accuracy in the validation dataset was 0.67 with a sensitivity of 0.85 and a specificity of 0.5.

**Conclusions:**

Although some imaging criteria suggest the diagnosis of GSC or GBM, differentiation between these two tumour entities on standard MRI alone is not feasible.

## Introduction

Gliosarcoma (GSC) is a rare IDH-wildtype variant of glioblastoma (GBM) accounting for approximately 1.8 to 8% of all glioblastomas.^1–3^ The entity is defined by the coexistence of glial and mesenchymal components. The glial pattern shows the typical features of GBM, whereas only the demonstration of a malignant mesenchymal component distinguishes GSC histologically from GBM. In addition to primary GSC, secondary GSC can occur after resection and radiotherapy of a GBM or as a radiation-induced tumor.^4^ Management and therapy is similar to that of GBMs with surgical resection and adjuvant radiochemotherapy.^3,5,6^ Metastatic disease has been reported in GSC.^7^ Outcome and prognosis, however, seems to be worse in GSC compared to GBM,^8–14^ which raises the question whether GSC should be treated more aggressively.

The radiological phenotype of GSC can mimic GBM or anaplastic meningioma^15–17^ as the main differential diagnoses. Owing to the rarity of the disease as compared to GBM, similar location and heterogeneous imaging characteristics, the preoperative diagnosis on the basis of imaging features alone is challenging, but it would be highly desirable to develop specific therapeutic approaches.

Several case series tried to determine the imaging characteristics of GSC.^15,16,18–20^ Predilection of the temporal lobe, peripheral location and involvement of the meninges with moderate to marked surrounding oedema have been described as typical features. GSCs located deep within the brain parenchyma, however, are even more difficult to distinguish from GBM. One study specifically compared imaging features in 48 GSC and 48 matched GBM patients and analyzed their discriminative power with a focus on Visually Accessible Rembrandt Images (VASARI) analysis.^20^ It found no singular characteristic or pathognomonic feature for GSC but reported a thicker enhancing tumour wall, often with a so-called paliform pattern, a higher rate of haemorrhage and an eccentric cystic portion in these lesions. In univariate analysis GSC tended to be larger than GBM with more enhancement, cortical involvement, less necrosis, a lower risk of ependymal invasion and a lower incidence of midline-crossing oedema. The authors called for further data to better understand the discriminatory power of neuroimaging.

The present study aims to analyze multiple imaging features on MRI of histologically proven GSC in comparison with an age- and sex-matched cohort of GBM and to develop and validate a multivariate logistic model to distinguish the two entities, refining previous univariate approaches.

## Materials and methods

This study was approved by the institutional ethical review board and conducted according to the principles of the Declaration of Helsinki. Owing to the retrospective character of data collection and analysis, written informed consent was waived.

### Patient selection

**Figure 1.**
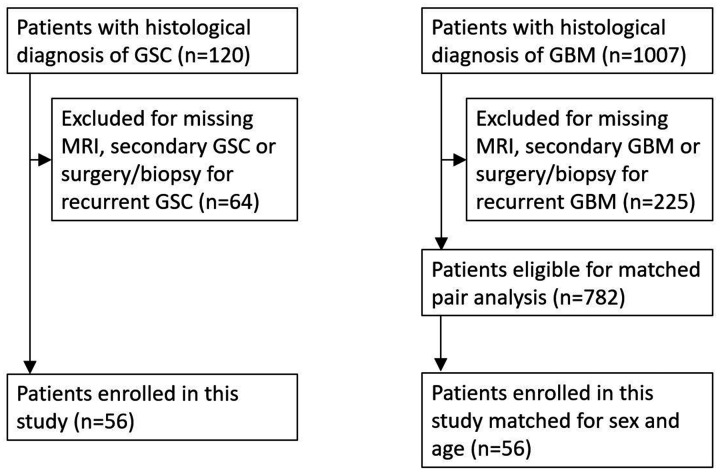
Study recruitment. GSC: gliosarcoma; GBM: glioblastoma; MRI: magnetic resonance imaging.

In this retrospective study we searched the electronic database of the departments of pathology at two centres for histologically proven GSC between January 1998 and December 2016. Differentiation of glioblastoma from gliosarcoma was performed by histopathology with dense reticulin fibre networks in significant parts of the tumour, not attributable to growth into the leptomeninges, being the major criteria for the diagnosis of gliosarcoma. In total, 120 histology reports were identified. Patients with recurrent GSC, secondary GSC (developing after radiation therapy of GBM) and patients without presurgical MRI were excluded. Fifty-six patients with histological proven GSC were available for analysis. The pathological databases were searched for GBM during the same time period and 1007 cases were identified. Patients with recurrent or secondary GBM and patients without presurgical MRI were omitted from this dataset. The 782 remaining patients were matched for age and sex with the GSC patients, and 56 GBM patients were identified for further analysis. The study recruitment process is shown in Figure 1.

### MR imaging analysis

MRI was performed with various scanners over the long study period. All imaging studies included T1-weighted images with and without contrast enhancement, T2-weighted images,  fluid-attenuated inversion recovery (FLAIR), DWI in 77% (n=86) and PWI (perfusion weighted imaging) in 35% (n=39). Loss of signal within the tumour on susceptibility weighted images (SWI) or T2*-weighted gradient echo sequences (GRE) was considered haemorrhage. In cases of several imaging studies, the latest study before surgery was used. A neuroradiologist (CJM) with 10 years of experience evaluated MR features for both tumour entities.

Based on the available literature of imaging features of GSC,^15–18,20,21^ we selected 24 imaging features for final analysis described in detail in Table 1.

**Table 1.** Description of radiographic features and Visually Accessible Rembrandt Images (VASARI) coding, if available.

Feature	Description	VASARI feature
Location	Frontal, temporal, insular, parietal or occipital lobe, brainstem, cerebellar	f1
Side	Right, bilateral or left	f2
Cyst	Presence of a non-necrotic cystic component	f8
Multifocal	Monofocal, multifocal or multicentric disease	f9
Diameter	Widest diameter of the contrast enhancing parts as measured on axial images in mm	n/a
T1/FLAIR ratio	Size of pre-contrast T1 hypointensity compared to approximate size of FLAIR abnormality (expansive, mixed, infiltrative)	f10
Thickness of enhancing margin	Maximal thickness measured on axial images in mm	n/a
Definition of enhancing margin	Well-defined, poorly defined	f12
Definition of non-enhancing margin	Well-defined, poorly defined	f13
Thickness of perifocal oedema	Maximal thickness measured on axial images in mm	n/a
Oedema/tumour ratio	Ratio between thickness of perifocal oedema and tumour diameter	n/a
Oedema crosses midline	Yes/no	n/a
Haemorrhage	Yes/no	f16
Diffusion characteristics	Facilitated/restricted/mixed	f17
ADC ratio	Ratio of ADC value (solid tumour) compared to contralateral region of interest at same location	n/a
ADC absolute value	Mean ADC value of solid tumour	n/a
Pial invasion	Enhancement of the overlying pia contiguous to enhancing or non-enhancing tumour matrix	f18
Ependymal invasion	Invasion of any adjacent ependymal surface contiguous to enhancing or non-enhancing tumour matrix	f19
Cortical involvement	Non-enhancing or enhancing tumour extending to the cortex, or cortex no longer distinguishable from subjacent tumour	f20
Deep white matter invasion	Enhancing or non-enhancing tumour extending into the internal capsule, corpus callosum or brainstem	f21
Tumour crosses midline	Enhancing tissue crosses into contralateral hemisphere through white matter commissures (excluding herniation)	f23
Satellites	Area of enhancement within the region of signal abnormality surrounding the dominant lesion but not abutting any part of the major tumour mass	f24
Calvarial remodelling	Erosion of inner table of skull	f25
CBV	Normal / elevated	n/a
CBV ratio	Mean CBV compared to contralateral region of interest at same location	n/a
Dural involvement	Contact of enhancing or non-enhancing tumour with or enhancement of the overlying dura	n/a

ADC: apparent diffusion coefficient; CBV: cerebral blood volume; n/a = not applicable.

### Statistical analysis

Statistical analyses were performed with R version 3.6.2 (The R Project for Statistical Computing; http://www.r-project.org/). The primary endpoint was histological diagnosis, GSC or GBM.

### Univariate analysis

Binary features were evaluated using odds ratio (OR) and Fisher’s exact test, categorical variables using OR and logistic regression. Odds ratios and 95% confidence intervals (CI) were calculated. Continuous variables were analyzed using the area under the receiver operator characteristics curve (AUC) to assess overall discriminatory power.

### Multivariate analysis

Missing data were imputted using the rfPermute package.^22^ For continuous variables, the weighted average of the non-missing observations was used for imputation, where the weights were the proximities. For categorical predictors, the imputed value was the category with the largest average proximity. To select variables for final analysis we used the importance measures of the random forest algorithm from the randomForest package.^23^ The features with the highest values were selected for the final model.^24^ Penalized likelihood estimation for the logistic regression analysis was performed using the least absolute shrinkage and selection operator (LASSO) method to avoid overfitting.

We assessed the predictive performance of the final model by examining discrimination based on the area under the curve (AUC) of the receiver-operating characteristic (ROC) curve and by examining calibration based on agreement between predicted and actual tumour type using a published dataset of VASARI features for GSC and GBM.^20^

## Results

The analysis compared 56 GSC patients with 56 age- and sex-matched GBM patients, 43% (*n*=24) of whom were female. Median age was 62 years ± 12.8 ranging from 32 to 85 years (IQR=58–73). Metastases outside the brain were not found in either gliosarcomas or glioblastomas. PWI results were available in 11 GSC and 28 GBM and showed relative hyperperfusion in all cases. Results of the univariate analysis of binary and categorical variables are presented in Table 2. Haemorrhage showed a clear association with GSC (OR = 2.89, *p* = 0.01). Interestingly, the features cyst (OR = 0.21, *p* < 0.01), pial invasion (OR = 0.07, *p* < 0.01), ependymal invasion (OR = 0.23, *p* < 0.01), multifocal or multicentric disease (OR = 0.82, *p* < 0.01) and definition of non-enhancing border (OR = 0.90, *p* = 0.02) showed significant associations with GBM histology. The infinite OR for calvarial remodelling with a p-value of 0.12 is due to the low incidence of only two cases. Of note, the preference of a certain lobe, e.g. temporal lobe, was not significant with an OR of 0.99 (95% CI = 0.91–1.08) and a *p*-value of 0.87, neither was dural involvement with an OR of 0.49 (95% CI = 0.19–1.23) and a *p*-value of 0.14. Of the quantitative features (Table 3) the thickness of perifocal oedema and the ratio oedema/tumour showed an AUC of 0.701 and 0.662, respectively, with GSC being associated with a greater thickness of the perifocal oedema absolutely and expressed as ratio. Contrast enhancing border and diameter showed a worse AUC of 0.599 and 0.603 with a *p*-value of 0.03 and 0.04, respectively.

**Table 2.** Univariate analysis for binary and categorical features with odds ratio, lower and higher 95% confidence interval (CI) and *p*-value.

	Odds ratio	95% CI		*p*-value
Cyst	0.21	0.085	0.491	<0.01
Defined border	0.15	0.003	1.333	0.11
Midline-crossing oedema	0.70	0.244	1.953	0.49
Haemorrhage	2.89	1.242	6.956	0.01
Pial invasion	0.07	0.021	0.184	<0.01
Ependymal invasion	0.23	0.094	0.531	<0.01
Cortical invasion	1.80	0.623	5.541	0.33
Deep WM invasion	0.40	0.102	1.380	0.18
Midline-crossing	0.35	0.074	1.315	0.09
Satellites	0.60	0.214	1.602	0.36
Remodelling	Inf	0.675	Inf	0.12
Lobe	0.99	0.91	1.08	0.87
Side	0.96	0.87	1.05	0.38
Multifocal	0.82	0.73	0.92	<0.01
Non-CE border	0.90	0.82	0.98	0.02
Diffusion↓	1.05	0.93	1.18	0.44
Dural involvement	0.49	0.19	1.23	0.14

CE: contrast enhancing; WM: white matter.

**Table 3.** Univariate analysis for continuous variables with area under the curve (AUC), lower and higher 95% confidence interval (CI) and *p*-value.

	AUC	95% CI		*p*-value
Diameter	0.599	0.492	0.706	0.04
CE border	0.603	0.496	0.709	0.03
Border	0.555	0.448	0.663	0.16
Oedema	0.701	0.603	0.799	<0.01
Oedema/tumour	0.662	0.561	0.764	<0.01
ADC ratio	0.549	0.426	0.671	0.22
ADC absolute	0.536	0.408	0.663	0.72

CE: contrast enhancing; ADC: apparent diffusion coefficient.

### Multivariate analysis with development of the logistic regression model

**Figure 2.**
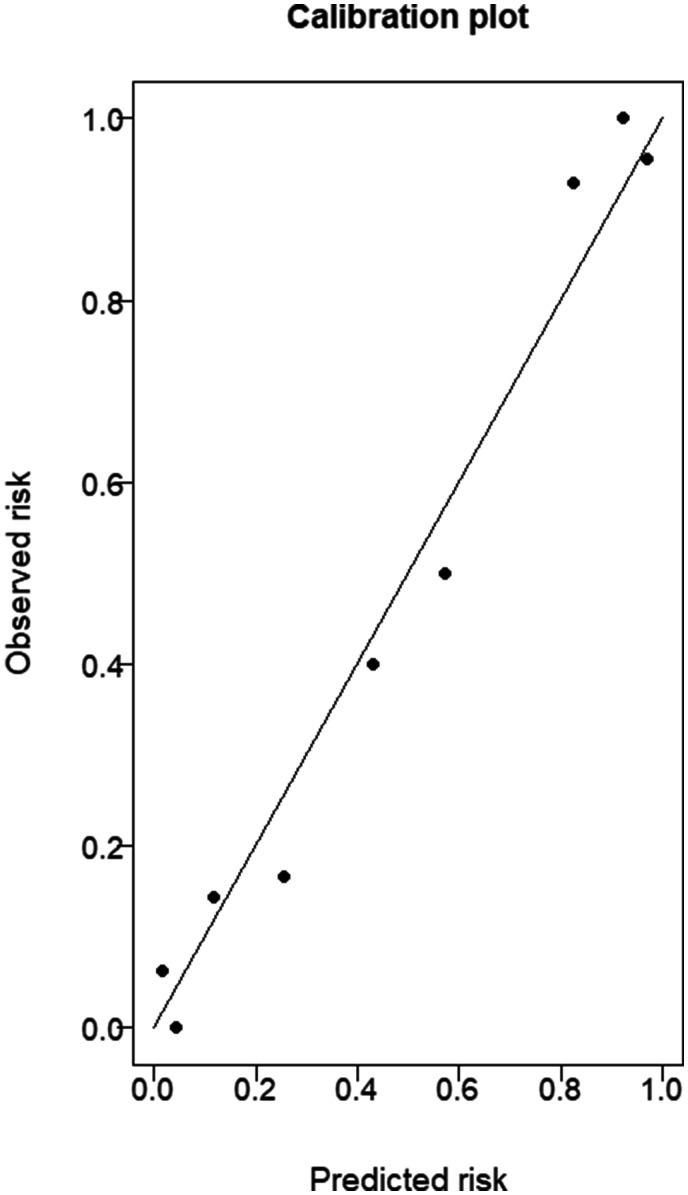
Calibration plot for the training dataset.

Variable importance was measured using the random forest method. Gini coefficients were calculated and the sample inbag rates were determined. The following variables were used for developing the logistic regression model: pial invasion, oedema, ependymal invasion, cyst, multifocal disease, definition of enhancing margin, haemorrhage and ratio oedema/tumour. After penalized likelihood ratios were estimated for the logistic regression analysis, the following parameters – all VASARI features – were included in the final model: presence of a cyst, pial invasion, haemorrhage and ependymal invasion. Table 4 shows the results of the logistic regression analysis for this final model. The calibration curve showed good agreement in the training dataset (Figure 2). The final model yielded a mean AUC of 0.919 on the training dataset and 0.746 on the validation dataset. The accuracy in the validation dataset was 0.67 with a sensitivity of 0.85 and a specificity of 0.5.

**Table 4.** Results of the logistic regression model.

	OR	95% CI		*p*-value
Cyst	0.13	0.03	4.01	<0.001
Pial invasion	0.03	0.01	1.04	<0.001
Haemorrhage	14.65	3.92	7.26	<0.001
Ependymal invasion	0.15	0.04	4.71	<0.001
Observations	112			
AIC	87.39			
BIC	100.98			

AIC: Akaike-Information-Criterion; BIC: Bayesian-Information-Criterion.

## Discussion

**Figure 3.**
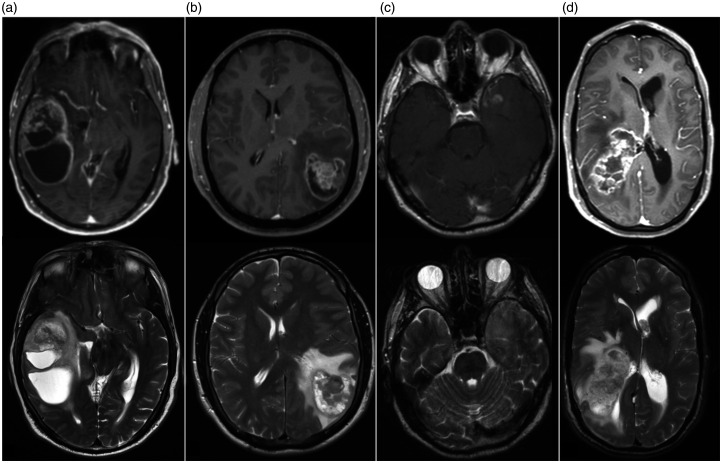
Imaging examples. Four patients with gliosarcoma (GSC) illustrating the spectrum of radiological phenotypes. Upper row shows contrast enhanced T1-weighted images, lower row the corresponding T2-weighted images. (a) Large tumour with typical GSC aspect in the temporal lobe, contact to the surface of the brain and large cystic components. (b) Peripheral, partial necrotic tumour without cystic component and pronounced oedema. (c) Small temporal tumour with only diffuse signal changes on T2-weighted – initially suspected grade 3 to 4 glioma. (d) large tumour associated with the right ventricle – classic glioblastoma aspect. All tumours were proven to be GSC on histological analysis.

Previous studies focused on the description of imaging features of GSC to determine certain specifics of this tumour entity and to discriminate GSC and GBM using univariate analysis. The multivariate model developed in this work tries to discriminate GSC and GBM on MR imaging. Still, only haemorrhage predicted GSC whereas pial and ependymal invasion and – opposing previous studies^20,21,25^ – the detection of a cystic component rather suggested GBM. We could not reproduce the association of dural involvement and predilection for the temporal lobe with GSC as a distinguishing feature from GBM suggested by other investigations;^1,17,26^ both did not predict GSC in our model. External validation with a well-studied dataset underscores the relevance of these results. We conclude that the discrimination of GSC and GBM is associated with a high error margin. This is illustrated by the four examples of GSCs shown in Figure 3.

Yi et al.^20^ analyzed 48 patients harbouring a GSC and 48 matched GBM patients with a partly overlapping set of variables and also found the association of GSC with haemorrhage, while ependymal invasion was related to GBM. The increased occurrence of cystic features could not be reproduced in our data. The results show that the use of SWI/GRE sequences for haemorrhage detection may be helpful for the diagnosis of GSC.

**Figure 4.**
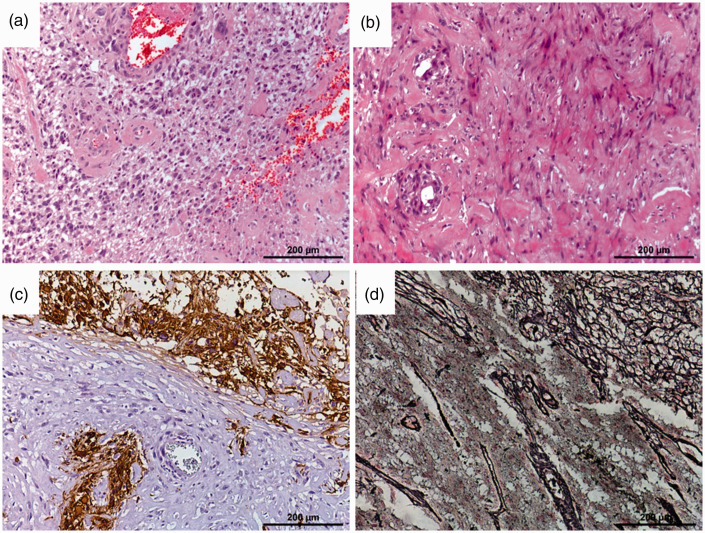
Histopathological features of both the glial and the mesenchymal components of gliosarcoma. (a) H&E stain of the glial compartment of a gliosarcoma with prominent vessels. (b) Mesenchymal compartment of the same tumour with collagenous fibres and spindle-cell-type nuclei. (c) GFAP (glial fibrillary acidic protein) immunohistochemistry staining the glial compartment, the mesenchymal compartment remains negative. (d) Silver stain (Tibor–Pap stain) shows dense reticulin fibre networks in the mesenchymal compartment (upper right), while the glial compartment is largely devoid of reticulin fibres (lower left).

Pathologically, GSC is a clearly defined tumour entity with stem cells that are able to differentiate into glial and mesenchymal components.^27^ The different components of GSC and the resulting histopathological polymorphism is demonstrated in Figure 4. The extent of the mesenchymal component varies significantly^21^ so that sampling errors have to be taken into account, particularly in cases where only biopsies or partial resections were performed.^28^ Several authors in the literature also raised the possibility of mis- or underdiagnosed secondary GSC after radiotherapy.^29–31^ Since the ratio of secondary transformation cases is unclear, the gold standard of histology is questionable.

Histologically, the sarcomatous part can express the pattern of spindle cell sarcoma, and other lines of mesenchymal differentiation have been described, e.g. formation of cartilage, bone, osteoid-chondroid tissue and muscle tissue or even lipomatous features.^32^ This polymorphism is mirrored in tumour morphology which might easily prevent the formation of homogeneous or distinct imaging characteristics. Adding to these qualitative features, quantitative variation of tumour parts plays an important role: the percentage of the mesenchymal component has been reported to correlate with improved survival time.^12,33^ The ratio of different components might also influence the radiological phenotype, and GBM can have atypical imaging features as well;^34^ however, a correlation of the extent or the subtype of the mesenchymal part with imaging parameters has not been established. The broad spectrum of possible cell differentiation in combination with the close relationship to GBM might explain our difficulties in discriminating the imaging characteristics of GSC and GBM.

In contrast to GBM and secondary GSC, primary GSC exhibits IDH(-) in molecular analysis and is therefore considered a wild-type GBM variant,^21,35^ which raises the fascinating possibility of identifying correlations of this molecular marker with imaging characteristics. Unfortunately, Peckham et al.’s analysis could not find a specific imaging pattern in their case series.^21^ However, advanced MRI imaging techniques may be able to determine the IDH status non-invasively in the future.^36^

There are several limitations to our study. First, this is a retrospective analysis of patients in just two centres over a long period of time, which accounts for a large variety of different MR scanning techniques and protocols with implications for imaging quality and analysis. We aimed to analyze imaging features of standard MRI sequences in order to make the results applicable in daily practice. Second, MR reading was performed by only one neuroradiologist, unblinded to histological diagnosis which might lead to observer or confirmation bias. The third limitation lies in the low number of 56 cases, only slightly offset by our attempt at external validation of results. Still, our cohort represents the largest cohort for imaging features of GSC on MRI and is not only a descriptive case series but the first multivariate model to explicitly focus on differentiation between GSC and GBM.

## Conclusion

We developed a multivariate logistic regression model to differentiate GBM and GSC by imaging features on standard MRI sequences with only poor accuracy on external validation. The broad spectrum of histological differentiations and the close histological, molecular and genetic relationship to GBM in combination with the rarity of the disease prevents a definite diagnosis based on standard imaging criteria.
